# Intraoral soft tissue lipomas: clinicopathological features from 91 cases diagnosed in a single Oral Pathology service

**DOI:** 10.4317/medoral.24023

**Published:** 2020-08-27

**Authors:** Fábio Ramôa Pires, Lucas Souza, Rafael Arruda, Marília Heffer Cantisano, Bruna Lavinas Sayed Picciani, Teresa Cristina Ribeiro Bartholomeu dos Santos

**Affiliations:** 1 DDS, PhD, Professor, Oral Pathology, School of Dentistry, State University of Rio de Janeiro, Rio de Janeiro/RJ, Brazil; 2 Undergraduate student, School of Dentistry, State University of Rio de Janeiro, Rio de Janeiro/RJ, Brazil; 3 DDS, PhD, Professor, Stomatology, School of Dentistry, State University of Rio de Janeiro, Rio de Janeiro/RJ, Brazil; 4 DDS, PhD, Professor, Stomatology, Fluminense Federal University, Nova Friburgo/RJ, Brazil; 5 DDS, MSc, Professor, Oral Pathology, School of Dentistry, State University of Rio de Janeiro, Rio de Janeiro/RJ, Brazil

## Abstract

**Background:**

intraoral soft tissue lipomas are relatively uncommon mesenchymal neoplasms. Few papers have been published comparing the clinicopathological features of these tumors in different populations. The aim of the present study was to analyze the clinicopathological features from intraoral soft tissue lipomas diagnosed in a Brazilian population.

**Material and Methods:**

all cases diagnosed as intraoral soft tissue lipomas in an Oral Pathology laboratory from 2005 to 2019 were retrieved and descriptively analyzed; statistical analysis was performed for comparison of the clinical and demographic parameters.

**Results:**

91 intraoral lipomas were retrieved, including 56 lipomas, 30 fibrolipomas, 2 spindle cell lipomas, 2 angiolipomas, and 1 chondrolipoma. Mean age of the patients was 62.2 years and females represented 57.1% of the sample. Mean time of complaint was 45.4 months and mean size of the lesions was 16.2 millimeters. Buccal mucosa (38.8%), lower lip (18.8%) and tongue (16.5%) were the most commonly affected locations. Fibrolipomas were more common in females (*p*=0,037) and presented as smaller lesions (*p*=0,011) in comparison to lipomas.

**Conclusions:**

report of clinicopathological data from intraoral lipomas aid in establishing their differential diagnostic criteria and clinical profile in this specific location.

** Key words:**Lipoma, fibrolipoma, oral, mouth, spindle cell lipoma, angiolipoma, chondrolipoma.

## Introduction

Lipomas are benign neoplasms originated from adipose tissue and are considered relatively common in several anatomical regions. About 15% to 20% of all lipomas affect the head and neck region and it is estimated that only 1 to 4% of these affect the oral cavity, where they represent 0.1 to 5% of all benign tumors (1-14). Etiology of lipomas remains uncertain, but hereditary and endocrine factors, trauma and infections have been postulated (1-14).

The term "lipoma" comprises several different entities, including the conventional lipoma and several other histological subtypes with different clinical and histological characteristics, such as: fibrolipoma, myxoid lipoma, angiolipoma, myolipoma, spindle cell lipoma/pleomorphic lipoma, infiltrating (intramuscular) lipoma, intermuscular lipoma, hibernoma, lipomatosis, lipoblastoma/lipoblastomatosis, salivary gland lipomas (sialolipomas), atypical lipomas (atypical lipomatous tumors), osteolipoma, chondrolipoma, angiomyolipoma, myelolipoma, and chondroid lipoma (1-14).

Most of these subtypes have been described and studied in subcutaneous tissue and other anatomical regions, and there is few information on the frequency and the clinicopathological features from these histological variants in the oral cavity. Moreover, comparison of data derived from different populations helps to understand their specific demographic and clinical profile. Therefore, the aim of the present study was to analyze the clinicopathological features from a series of intraoral lipomas diagnosed in a Brazilian population over a 15-year period.

## Material and Methods

The files of the Oral Pathology laboratory, School of Dentistry, State University of Rio de Janeiro, Rio de Janeiro/RJ, Brazil, were reviewed from 2005 and 2019 and all cases diagnosed as lipomas and their subtypes were retrieved. Cases diagnosed through fine-needle aspiration biopsy, cases not located in the oral cavity (eg. submandibular area) and cases presenting no histological slides and/or paraffin blocks for review were excluded from the sample. Demographic and clinicopathological information were retrieved from the patient´s registries and included: gender; age; location and clinical features of the lesions; time of onset; and final diagnosis. All hematoxylin and eosin (HE)-stained histological slides were carefully reviewed and lipoma subtypes were classified according to reference literature on the field (1-14). When applicable, immunoreactions were performed with antibodies directed to mast cell tryptase (clone AA1, Dako Cytomation, Glostrup, Denmark, dilution 1:10.000) and CD34 (Biocare Medical, Concord, US, dilution 1:500) for spindle cell lipomas. Data were descriptively analyzed for all cases and each of the variants and statistical analysis (Pearson chi-square and t test) was performed for comparison of the clinical characteristics from lipomas and fibrolipomas, with statistical significance considered when *p*<0,05.

## Results

**Figure 1:**
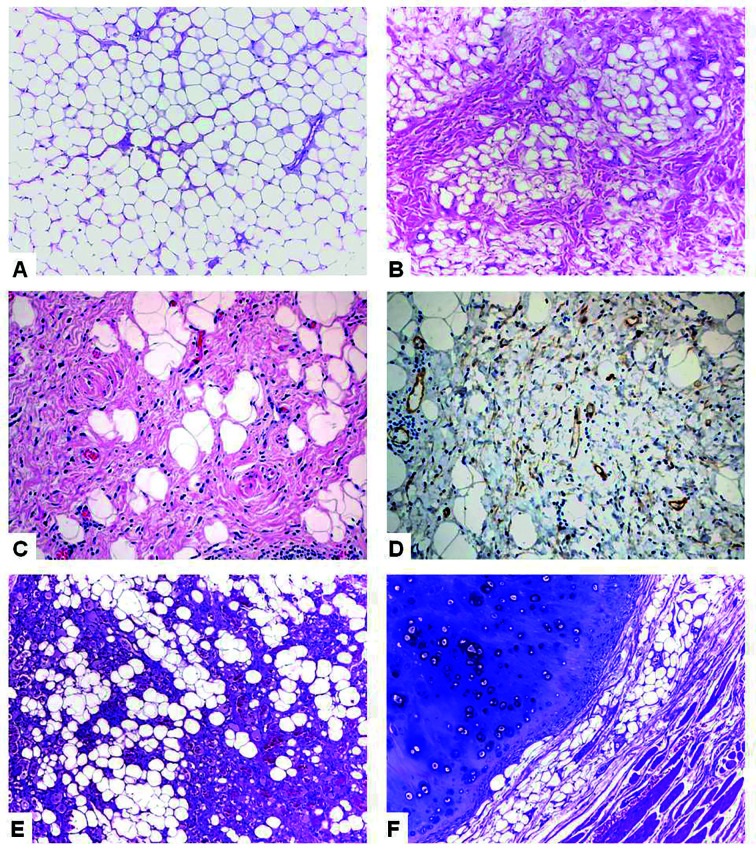
Histological characteristics of intraoral lipomas. A. Conventional lipoma showing a monotonous proliferation of mature adipocytes (HE, 10x); B. Presence of mature adipocytes and a collagenous fibrous tissue in fibrolipoma (HE, 10x); C. Spindle cells and adipocytes in a spindle cell lipoma (HE, 40x); D. CD34-positive spindle cells in spindle cell lipoma (Immunoperoxidase, 40x); E. Adipose tissue associated to fibrin thrombi-containing small blood vessels in angiolipoma (HE, 10x); F. Mature chondroid tissue associated with adipose tissue in chondrolipoma (HE, 10x).

The final sample was composed by 91 intraoral lipomas, representing 0.68% of the 13.392 registries in the period. These included 56 conventional lipomas (61.5%), 30 fibrolipomas (33%), 2 spindle cell lipomas (2.2%), 2 angiolipomas (2.2%) and 1 chondrolipoma (1.1%) (Fig. 1). Considering all subtypes as a group, females and males were affected in, respectively, 52 (57.1%) and 39 cases (42.9%). Mean age of the affected patients was 62.2 years (ranging from 27 to 95 years) and mean age of females (61.4 years) and males (63.3 years) were similar (*p*=0,521). Mean time of onset was 45.4 months (ranging from 1 to 360 months) and the mean time reported by females (41.7 months) and males (55.7 months) showed no statistical significant difference (*p*=0,559).

The exact location of the lesions was reported in 85 cases and showed that they were located in the buccal mucosa (33 cases, 38.8%), lower lip (16 cases, 18.8%), tongue (14 cases, 16.5%), mucobuccal fold (9 cases, 10.6%), palate (6 cases, 7.1%), floor of mouth (4 cases, 4.7%) and alveolar mucosa (3 cases, 3.5%). Most lesions affecting females were situated in the buccal mucosa (46%), lower lip (20%), and tongue (12%), while in males the most common locations were buccal mucosa (29%), tongue (23%), and lower lip (17%) (14%) (*p*=0,417). Mean size of the lesions was 16.2 mm (ranging from 1 to 60 mm) and it was similar when comparing females (15.4 mm) and males (17.4 mm) (*p*=0,508). Base of the lesions was described in 44 cases and showed that most were sessile (29 cases, 65.9%), and there were no statistically significant differences according with the different affected locations (*p*=0,473) or final histological diagnosis (*p*=0,060).

Clinical diagnosis of the 91 cases included lipoma/fibrolipoma (60 cases, 66%), fibrous hyperplasia (39 cases, 43%), other reactive conditions (9 cases, 10%), other benign neoplasms (4 cases, 4%), soft tissue cysts/pseudocysts (5 cases, 5%) and other entities (2 cases, 2%). When considering only the 56 lipomas, clinical diagnosis included mostly lipoma/fibrolipoma (45 cases, 80%), fibrous hyperplasia (14 cases, 25%), other benign neoplasms (3 cases, 5%) and soft tissue cysts/pseudocysts (3 cases, 5%); for the 30 fibrolipomas, clinical diagnosis included mostly fibrous hyperplasia (24 cases, 80%), lipoma/fibrolipoma (13 cases, 43%) and other reactive conditions (5 cases, 17%).

Comparative analysis of the anatomical location of the lesions according with final histological diagnosis showed statistical significant differences when comparing lipomas and fibrolipomas (*p*=0,043). Lipomas presented no predilection for gender, but fibrolipomas preferentially affected females (73%) (*p*=0,037). There were no statistically significant differences when comparing mean age of affected patients (*p*=0,904), mean time of onset (*p*=0,932) and base of the lesions (*p*=0,074) when comparing lipomas and fibrolipomas. In contrast, mean size of lipomas (18.7 mm) was greater than mean size of fibrolipomas (10.7 mm) (*p*=0,011) (Table 1).

**Table 1:**
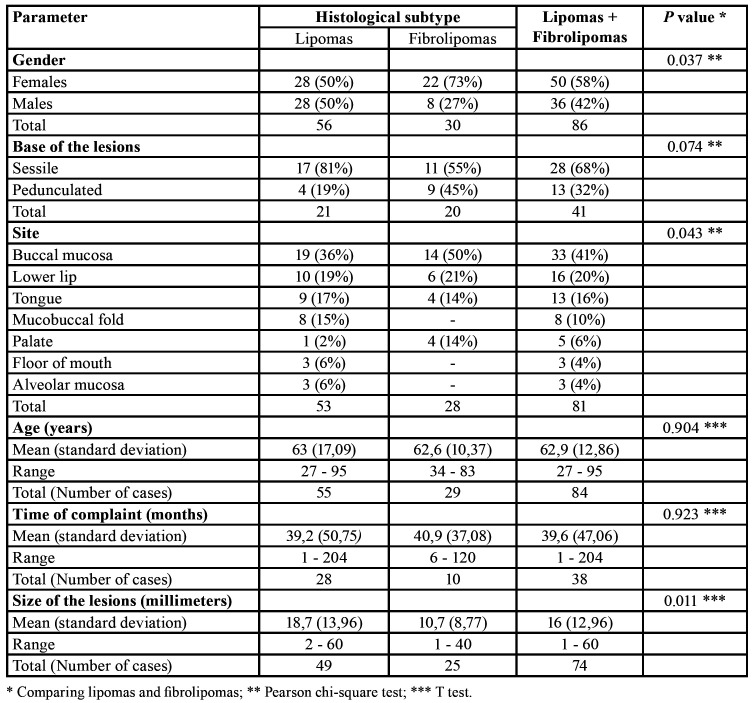
Comparison of demographic and clinical parameters from intraoral lipomas and fibrolipomas from the studied sample.

Spindle cell lipoma was diagnosed in 2 patients, including a 360-month lasting swelling in a 50-year-old male (location was not precise) and a 24-month swelling in the floor of the mouth of a 45-year-old female. Angiolipomas were diagnosed in a 12-month lasting 15 mm pedunculated swelling in the palate (pterygomandibular raphe) of a 52-year-old male, and in a 4-month lasting 45 mm submucosal nodule in the mucobuccal fold of a 28-year-old female. These two cases were previously reported (15). Chondrolipoma was diagnosed in a 10 mm swelling in the tongue of a 82-year-old male.

## Discussion

In general, lipomas represent from 0.12% to 4.4% of all lesions diagnosed in Oral Pathology Laboratories (1-14), in accordance with our results. Intraoral lipomas and their subtypes usually present as submucosal mobile yellowish to pinkish soft to fibrous swellings, covered by normal oral mucosa. They typically present as long-lasting growths and mean onset varies from 28 to 76 months, ranging from 15 days to 43 years (1,3,4,5,7,10,12,14). Mean size ranges from 1.1 to 3.0 cm and they are virtually always asymptomatic (1,4,5,7,8,10,12,14). Although there seems to be no gender predilection for intraoral lipomas, most studies show a female (1,3,5,6-8,12,13) rather than male (2,4,9,11) predilection. Mean age of the affected patients is usually on the 6th to 7th decades of life and most patients are in their fourties to sixties (1,3-12,14). The buccal mucosa (31 to 66% of the cases), tongue (10 to 31%), mucobuccal fold (3 to 30%), lower lip (8 to 21%), floor of mouth (5 to 22%) and retromolar area and alveolar mucosa (1 to 12%), are the most commonly affected areas for intraoral lipomas (1-13). The results of the present study reinforce these findings.

Although these general characteristics can be applied to most intraoral lipomas, several lipoma subtypes have been reported in the oral cavity with variable frequencies and, due to their rarity, few information is presently available (Table 2) (1-14). Apart from conventional lipomas (the most common subtype, representing 36 to 83% of the cases), other histological variants including fibrolipomas (1 to 40%), intramuscular lipomas (2 to 17%), spindle cell lipoma (1 to 44%), sialolipoma (2 to 10%), myxoid lipoma (up to 5%), angiolipoma (1 to 4%), osteolipoma (1 to 2%), chondrolipoma (1 to 2%) and atypical lipomatous tumor (up to 9%) have been reported in the literature (1-14). Cytogenetic analysis has shown several different chromosomal alterations in some lipomatous tumors (10), but, in clinical practice, their diagnosis remain based on conventional HE features, eventually associated to specific immunoreactions. For this reason and, as shown by the variable frequency of the histological subtypes reported in the published series, it is essential to follow very strict diagnostic criteria when classifying these tumors, allowing reliable comparisons on their frequencies, morphological patterns, clinical features and biological behavior.

**Table 2:**
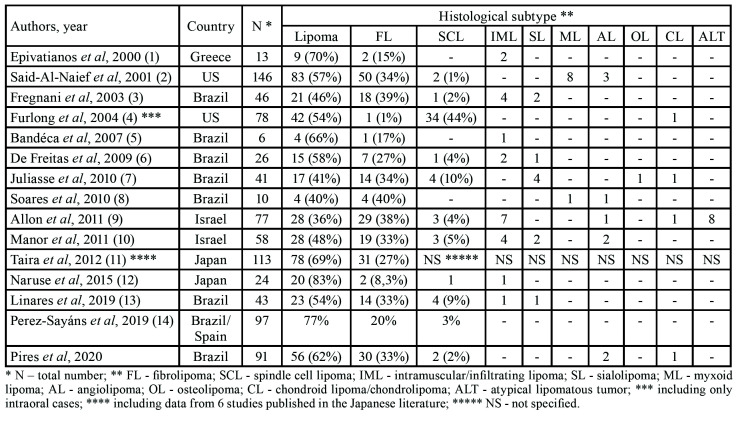
Relative frequency of intraoral lipomatous tumors in the most representative series reported in the literature since 2000.

Conventional lipomas are characterized by a proliferation of mature adipocytes interspersed by variable amounts of connective tissue containing bundles of collagen fibers and blood vessels (4). Despite these tumors rarely present a fibrous capsule, the proliferation of adipocytes shows a well-defined limit to the adjacent normal tissue. The term fibrolipoma is used when, apart from the typical adipocyte proliferation seen in a conventional lipoma, dense fibrocollagenous connective tissue bands are interlacing the mature adipocytes (2). There are no strict criteria to define whether the amount and distribution of fibrous tissue is sufficient to define the lesion as a fibrolipoma, and this seems to explain the variable lipoma:fibrolipoma ratios reported in the literature (1,2,4). Most studies have shown that conventional lipomas are more common than fibrolipomas, except for one study where fibrolipomas (38%) outnumbered conventional lipomas (36%) (9), probably due to the histological criteria applied more than by a true geographic and/or populational variation. Apart from that, it is essential to rule out other similar lesions, such as herniated adipose tissue and fibrous hyperplasias with fat entrapment, when diagnosing these two histological subtypes (3). One interesting feature is that fibrolipomas seem to be more common in females (5,11,12,14,16), in accordance with the present results.

Myxoid lipoma has been considered a subtype of lipoma characterized by the presence of a myxoid background associated to and partially replacing the proliferative adipose tissue (2,8). As conventional lipomas and spindle cell lipomas can also present myxoid areas, it is essential to rule out these two histological subtypes before rendering a diagnosis of myxoid lipoma. None of the intraoral lipomas reported in the present study presented an exclusive myxoid background and, for this reason, none was classified as myxoid lipoma. More comparative studies are necessary to define if myxoid lipoma is a true histological subtype of lipomas or if these lesions are characterized by myxoid degenerative connective tissue changes. Supporting this controversial issue, Furlong *et al*. (4) have reported that 11 of their cases previously diagnosed as myxolipoma were classified as spindle cell lipoma with prominent myxoid change after revision.

Intramuscular (or infiltrative) lipomas are slow-growing lesions usually found in the extremities, thigh and trunk of adults (4). This variant is characterized by the presence of mature adipocytes showing no atypia infiltrating adjacent muscle layers and, especially in the extremities, these lesions can show infiltrative growth and higher recurrence rates. Intraoral intramuscular lipomas have been reported, but in this specific anatomical location, they usually show a biological behavior and a proliferative activity similar to conventional lipomas (1,3,7). Other subtypes of intraoral lipomas can show this close relationship to muscle fibers, suggesting that this feature should be interpreted mostly as a consequence of anatomical proximity, without changing the typical benign and indolent behavior of intraoral lipomas (5,7). To reinforce this anatomical relationship, most intraoral intramuscular lipomas have been reported in the tongue, where muscle fibers are in close superficial contact with the adipose tissue (1,3,4,10). For this reason no intramuscular lipomas were diagnosed in the present study. However, even with these evidences, it is worthwhile to consider that any infiltrating oral adipocytic tumor should be carefully analyzed to rule out the possibility of oral liposarcoma. In the latter, the presence of pleomorphic cells with nuclear hyperchromatism and vacuolization, as well as lipoblasts and higher mitotic activity can aid diagnosis, although they can be only focally present (1,10).

Chondroid lipomas are characterized by the presence of mature adipose tissue, lipoblast-like cells and a chondroid matrix, and should be differentiated from liposarcomas and chondrosarcomas (10). This entity should be differentiated from chondrolipoma, as in the latter there is mature cartilaginous metaplastic tissue, probably secreted by pluripotential mesenchymal cells (10). This feature is similar to what occurs in osteolipomas, where metaplastic bone is formed (10). One chondrolipoma was found in the present sample, affecting the tongue of an 82-year-old male. Sialolipomas are characterized by the presence of adipocytic mature cells associated to atrophic salivary acini and dilated salivary ducts (3), but no case was found in the present series.

Angiolipoma usually affects the subcutaneous tissue of the upper limbs and trunk of young adult males, being frequently multiple and tender to painful at palpation (10). They show a proliferation of mature adipose tissue interspersed by small thin-walled blood vessels containing fibrin thrombi in their lumen. Few cases have been reported in the oral cavity and most affected the buccal mucosa and mucobuccal fold as painless solitary submucosal masses (8,10,15). It is essential to call attention that the presence of a higher amount of blood vessels associated to mature adipose tissue is not sufficient for diagnosis of angiolipoma, as the presence of fibrin thrombi is essential for diagnosis (15). The present series revealed two angiolipomas affecting the pterigomandibular raphe and mucobuccal fold of adults. Both cases were reported in detail elsewhere, with a discussion on their main clinicopathological features and differential diagnosis (15).

Spindle cell lipoma commonly arises as a painless slow-growing mass in the subcutaneous tissue from the neck and trunk of adult males. The first description of spindle cell lipoma was rendered by Enzinger and Harvey in 1975 (17), but McDaniel *et al*. in 1984 (18) were the first to report an intraoral spindle cell lipoma. This entity usually affects adults without gender predilection, being characterized by a submerse painless mass covered by normal mucosa that affects mostly the tongue, buccal mucosa and floor of the mouth (2,19-21). Although it has been considered that lipomas and fibrolipomas are the most common intraoral lipoma subtypes, Furlong *et al*. (4) reported that spindle cell lipoma represented 44% of their intraoral lipomas, affecting mostly the lips, buccal mucosa and tongue. This frequency probably do not reflect a true geographical or populational predilection but, as a reference center, a bias due to the referral of more complex cases to second opinion/revision. Curiously, none of the cases reported in the present study affected the buccal mucosa, also supporting that spindle cell lipomas present a slightly different site distribution in comparison to conventional intraoral lipomas and fibrolipomas.

Oral spindle cell lipoma is composed by variable amounts of mature adipocytes, CD34-positive spindle cells and mast cells in a background of myxoid and collagenous connective tissue (20,21). Spindle cells are characterized by a single elongated nucleus, bipolar cytoplasmic process and occasional cytoplasmic vacuoles (2,19). As previously discussed, it is possible that some spindle cell lipomas with prominent myxoid stroma were classified as myxoid lipomas in the past, and, also, more collagenous lesions could have been misinterpreted as fibrolipomas (4,7). These features call attention to the importance of CD34 immunostaining in the spindle cells (21). Although some spindle cell lipomas can show focal atrophic changes in adipocytes and focal atypia (including hyperchromatic nuclei and floret-like multinucleated cells), lipoblasts and widespread atypical features are not a feature and this is useful in differentiating these entities from oral atypical lipomatous tumors and well-differentiated liposarcomas (9). The reported features of focal atypia found in spindle cell lipomas were mostly associated to another lipoma subtype, called pleomorphic lipoma, which is presently considered as closely associated to spindle cell lipoma, being part of the same spectrum. The spindle cells are, as a rule, positive to CD34 and vimentin, and negative for most other markers, such as S100 protein, alpha smooth muscle actin, desmin and factor VIII (2,7,19-21). It is also important to highlight that focal chondroid changes can be found in spindle cell lipomas and should be taken in account when diagnosing these entities (21). Fregnani *et al*. (3) have shown that intraoral spindle cell lipoma shows a higher proliferative rate in comparison to conventional intraoral lipomas, but the biological behavior seems to be similar and no recurrences are expected after conservative surgical approach (2,3,19,20).

Although oral liposarcoma is an uncommon neoplasm, it is important to rule out this possibility when diagnosing oral adipocytic tumors (9,21,22,23). The term atypical lipomatous tumor has been used instead of well-differentiated liposarcoma for oral tumors presenting few lipoblasts and discrete infiltrative pattern, as in this location most lesions have an excellent prognosis, in contrast to well-differentiated liposarcomas elsewhere. Some atypical lipomatous tumors can show focal areas of stromal myxoid degeneration and, due to the discrete presence of lipoblasts and cellular atypia in most cases, they can be misinterpreted as myxoid lipomas. However, as this specific subtype has been a matter of controversy, it is important to consider other microscopic differential diagnosis, such as conventional lipomas with myxoid background or spindle cell lipoma, when dealing with these specific cases. Additionally, lipoblasts can be sparse in many atypical lipomatous tumors, reinforcing their resemblance to most conventional lipoma subtypes (22). For further confusion, oral liposarcomas have a very similar clinical profile than oral lipomas, mostly affecting adults and being characterized as long-lasting submerse nodules located especially in the tongue, buccal mucosa and floor of mouth (9,21-23). There were no cases diagnosed as atypical lipomatous tumor or liposarcoma in the present sample.

It seems that, considering the previously exposed data, there are several clinical and microscopic features that differ intraoral lipoma subtypes from their counterparts in other anatomical locations. For this reason, clinicopathological series including intraoral lipomas will offer the possibility of establishing these differential criteria and, consequently, determine the most appropriate diagnostic and management steps for these tumors when affecting this specific anatomical location.

## References

[1] Epivatianos A, Markopoulos AK, Papanayotou P (2000). Benign tumors of adipose tissue of the oral cavity: a clinicopathologic study of 13 cases. J Oral Maxillofac Surg.

[2] Said-Al-Naief N, Zahurullah FR, Sciubba JJ (2001). Oral spindle cell lipoma. Ann Diagn Pathol.

[3] Fregnani ER, Pires FR, Falzoni R, Lopes MA, Vargas PA (2003). Lipomas of the oral cavity: clinical findings, histological classification and proliferative activity of 46 cases. Int J Oral Maxillofac Surg.

[4] Furlong MA, Fanburg-Smith JC, Childers ELB (2004). Lipoma of the oral and maxillofacial region: site and subclassification of 125 cases. Oral Surg Oral Med Oral Pathol Oral Radiol Endod.

[5] Bandéca MC, de Pádua JM, Nadalin MR, Ozório JEV, Silva-Sousa YTC, Perez DEC (2007). Oral soft tissue lipomas: a case series. J Can Dent Assoc.

[6] de Freitas MA, Freitas VS, de Lima AA, Pereira FB Jr, dos Santos JN (2009). Intraoral lipomas: a study of 26 cases in a Brazilian population. Quintessence Int.

[7] Juliasse LER, Nonaka CFW, Pinto LP, Freitas RA, Miguel MCC (2010). Lipomas of the oral cavity: clinical and histopathologic study of 41 cases in a Brazilian population. Eur Arch Otorhinolaryngol.

[8] Soares ECS, Costa FWG, Sousa FB, Alves APNN, Osterne RLV (2010). Oral lipomas in a Brazilian population: a 10-year study and analysis of 450 cases reported in the literature. Med Oral Patol Oral Cir Bucal.

[9] Allon I, Aballo S, Dayan D, Vered M (2011). Lipomatous tumors of the oral mucosa: histomorphological, histochemical and immunohistochemical features. Acta Histochemica.

[10] Manor E, Sion-Vardy N, Joshua BZ, Bodner L (2011). Oral lipoma: analysis of 58 new cases and review of the literature. Ann Diagn Pathol.

[11] Taira Y, Yasukawa K, Yamamori I, Iino M (2012). Oral lipoma extending superiorly from mandibular gingivobuccal fold to gingiva: a case report and analysis of 207 patients with oral lipoma in Japan. Odontology.

[12] Naruse T, Yanamoto S, Yamada S, Rokutanda S, Kawakita A, Takahashi H (2015). Lipomas of the oral cavity: clinicopathological and immunohistochemical study of 24 cases and review of the literature. Indian J Otolaryngol Head Neck Surg.

[13] Linares MF, Leonel ACLS, Carvalho EJA, Castro JFL, de Almeida OP, Perez DEC (2019). Intraoral lipomas: a clinicopathological study of 43 cases, including four cases of spindle cell/pleomorphic subtype. Med Oral Patol Oral Cir Bucal.

[14] Perez-Sayáns M, Blanco-Carrión A, Oliveira-Alves MG, Almeida JD, Anbinder AL, Mendoza IL (2019). Multicentre retrospective study of 97 cases of intraoral lipoma. J Oral Pathol Med.

[15] Silva-Júnior GO, Picciani BL, Costa RC, Barbosa SM, Silvares MG, Souza RB (2013). Oral soft-tissue angiolipoma: report of two cases of rare oral lipomatous lesion with emphasis on morphological and immunohistochemical features. J Oral Sci.

[16] De Visscher JG (1982). Lipomas and fibrolipomas of the oral cavity. J Maxillofac Surg.

[17] Enzinger FM, Harvey DA (1975). Spindle cell lipoma. Cancer.

[18] McDaniel RK, Newland JR, Chiles DG (1984). Intraoral spindle cell lipoma: case report with correlated light and electron microscopy. Oral Surg Oral Med Oral Pathol.

[19] Billings SD, Henley JD, Summerlin DJ, Vakili S, Tomich CE (2006). Spindle cell lipoma of the oral cavity. Am J Dermatopathol.

[20] Lau SK, Bishop JA, Thompson LD (2015). Spindle cell lipoma of the tongue: a clinicopathologic study of 8 cases and review of the literature. Head Neck Pathol.

[21] Shon W, Billings SD (2020). Soft tissue special issue: selected topics in the pathology of adipocytic tumors. Head Neck Pathol.

[22] Nascimento AF, Mc Menamin ME, Fletcher CDM (2002). Liposarcomas/atypical lipomatous tumors of the oral cavity: a clinicopathologic study of 23 cases. Ann Diagn Pathol.

[23] Fanburg-Smith JC, Furlong MA, Childers ELB (2002). Liposarcoma of the oral and salivary gland region: a clinicopathologic study of 18 cases with emphasis on specific sites, morphologic subtypes, and clinical outcome. Mod Pathol.

